# Dual-branch information extraction and local attention anchor-free network for defect detection

**DOI:** 10.1038/s41598-024-61324-8

**Published:** 2024-05-13

**Authors:** Xiaobin Wang, Qiang Zhang, Chengjun Chen

**Affiliations:** School of Mechanical and Automotive Engineering, Qingdao University of Technology, Qingdao, 266520 Shandong China

**Keywords:** Defect detection, FCOS, Anchor-free, Local attention network, Electrical and electronic engineering, Information technology

## Abstract

In the production process, the presence of surface defects seriously affects the quality of industrial products. Existing defect detectors are not suitable for surface with scattered distribution and complex texture of defects. In this study, a dual-branch information extraction and local attention anchor-free network for defect detection (DLA-FCOS), which is based on the fully convolutional one-stage network, is proposed to accurately locate and detect surface defects of industrial products. Firstly, a dual-branch feature extraction network (DFENeT) is proposed and used to improve the extraction ability of complex defects. Then, a local feature enhancement module is proposed, and a residual connection is established to enrich local semantic information. Meanwhile, the self-attention mechanism is introduced to form local attentional residual feature pyramid networks (LA-RFPN) to eliminate the influences of feature misalignments. The mean average accuracy (mAP) and frames per second (FPS) of the proposed DLA-FCOS on the cut layer of the tobacco packet defect dataset (CLTP-DD) are 96.8% and 20.7, respectively, which meets the requirements for accurate and real-time defect detection. Meanwhile, the average accuracy of the proposed DLA-FCOS on the NEU-DET and GC10-DET datasets is 78.4% and 67.7%, respectively. The results demonstrate that the DLA-FCOS has good feasibility and high generalization capability to perform defect detection tasks of industrial products.

## Introduction

In large-scale industrial production processes, the presence of surface defects seriously affects the quality and appearance of products. Limited by the production environment and conditions, defects will inevitably occur in the manufacturing process, such as patches and crazing on the surface of steel strips, mould and varia in tobacco packaging, etc. These defective products not only reduce performance and quality but also bring economic losses to the manufacturer. Therefore, accurately identifying the surface defects of products has become an urgent issue. Over the past decades, vision-based detection methods have developed rapidly in industrial applications. Traditional machine vision-based detection methods usually involve two main steps: feature extraction and classification. These methods first extract features such as texture^1^ and spectrum^2–4^ and then employ classifiers like SVM^5^, ELM^6^, or clustering^7–9^ to perform detection tasks. However, these traditional methods heavily rely on manually designed algorithms^10^ and are mostly suitable for specific types of defects, so they suffer from poor robustness and generalization. Since the surface of objects (such as cut layers of tobacco packets) usually contains small defects with complex textures, it is challenging to design classifiers that can achieve the desired performance^11^.

With advances in computer technology, there is a growing interest in using convolutional neural networks (CNNs) for defect detection and fault diagnosis. These methods can be mainly divided into two categories: those that classify defect images based on extracted information^12,13^ and those that simultaneously determine the position and size of the defects during classification. The former typically uses deep belief networks to analyze images and obtain fault classifications. For example, Zhao et al.^14^ combined an improved convolutional deep belief network (CDBN) to process feature images and classify faults, and this method achieved significant improvement in bearing data sets compared to other traditional methods. The method of this class is effective for a single type of dataset but lacks generalization. The latter typically requires the integration of region-level object detectors such as YOLOv5^15^, SSD^16^, and RetinaNet^17^, and such methods have shown superior performance in defect detection tasks and have been widely used^18,19^. According to differences in anchor settings and prediction patterns, object detectors can be roughly categorized into two types: anchor-based and anchor-free methods. In recent years, anchor-based defect detectors have been improved^20–23^ to adapt to various detection tasks in different environments. For instance, Huang et al.^20^ introduced a coordinate attention mechanism into the YOLOv5 model and adopted a feature pyramid and pixel aggregation network fusion architecture to realize effective feature integration in solar panel defect detection. Cheng et al.^21^ incorporated deformable convolutions into the DS-Cascade RCNN to reduce background noise in feature maps and effectively detect hub defects. Liu et al.^22^ utilized a K-Means +  + clustering-based anchor generation algorithm and an improved DenseNet for overhead line defect data. Jiang et al.^23^ developed an improved SSD network that combines multi-scale and attention mechanisms for PCB defect detection, and it achieved an average precision of 97.3% on the test set, meeting industrial requirements for PCB equipment inspection. However, anchor-based detectors require a large number of anchors for predictions, resulting in redundant computations and high training costs. This leads to slower detection speeds and reduced generalization capabilities, thus limiting their applicability in defect tasks.

Anchor-free networks, which have emerged in recent years, can directly predict and regress targets using pixel points without requiring anchor setting. This approach reduces computational complexity while maintaining a balance between positive and negative samples. Given these advantages, scholars have begun to apply improved anchor-free object detectors to defect detection. For example, Long et al.^24^ introduced channel-fusion convolution into the fully convolutional one-stage (FCOS) network^25^ and proposed a new feature fusion network called TF-FPN, which achieved significantly higher accuracy in plastic packaging defect detection than other algorithms. Yu et al.^26^ introduced an anchor-free network with a channel attention mechanism (CAM) attention mechanism and Complete-IoU (CIoU) loss function, achieving an average accuracy of 76.68% on a hot-rolled steel surface defect dataset. However, anchor-free defect detectors, such as FCOS, still have certain defects, including unstable training results due to the absence of anchor boxes and feature misalignment issues during fusion, resulting in decreased detection accuracy. Meanwhile, for non-dense defects, applying dense prediction-based FCOS can lead to false positives. Therefore, it is necessary to make corresponding improvements to the network to meet the requirements of non-dense defect detection.

In this study, an FCOS-based improved detection algorithm called the dual-branch information extraction and local attention anchor-free network (DLA-FCOS) is proposed and used for the detection of surface defects in various industrial products. First, a lightweight and intricate environment feature extraction network called dual-branch feature extraction network (DFENet) is developed, which leverages multi-stage fusion techniques for defect feature extraction to further improve detection accuracy. Meanwhile, a feature fusion network called local attentional residual-feature pyramid network (LA-RFPN) is developed, which can address the issue of feature misalignment during the fusion process through localized feature enhancement and the utilization of an improved self-attention mechanism. In this way, false positives and false negatives can be reduced.

The contributions of this study are summarized as follows:

1. To realize defect detection on the surface of industrial products, an anchor-free defect detector called DLA-FCOS is developed for multiple types of defect detection tasks.
2. To further enhance detection accuracy, a new dual-branch feature extraction network called DFENet is developed.
3. A novel feature fusion network called LA-RFPN is proposed, which employs residual connections and convolutional networks to amplify local features. Instead of deformable convolution networks (DCN), the embedded bi-level routing attention, which is an improved self-attention mechanism, was employed to eliminate the feature information of misalignments.

The rest of this paper is organized as follows: Section "Related work" provides related work, Section "Method" elaborates on the overall architecture of the DLA-FCOS, Section "Experiments and results" presents experimental results on multiple defect datasets to validate the effectiveness of the proposed method, Section "Discussion" discusses the proposed method, and Section "Conclusion" gives the conclusions.

## Related work

### Anchor-free defect detection methods

Defect detection has been widely used in industrial production to identify and localize object defects to guarantee smooth operation and rational product quality. With the development of deep learning, CNNs have been widely used for defect detection. For instance, Kwon et al*.*^18^ presented a dual-model approach based on YOLO for detecting welding defects; He et al*.*^19^ designed an element-wise convolutional feature fusion network for the classification of defects in display panels.

CNN-based object detectors can be categorized into anchor-based detectors and anchor-free detectors. Anchor-based object detectors pre-generate anchors with different scales and aspect ratios for pixels. The positive and negative samples are determined by calculating the intersection over union (IoU) of the anchor boxes and the ground truth boxes, the losses are calculated, and the positions of the detected objects are corrected based on the regression offsets. Anchor-based detectors have been introduced earlier, and they have gained more research and application in defect detection. For instance, Huang et al*.*^20^ investigated defect detection in solar panels by using an improved YOLOv5; Cheng et al*.*^21^ reported enhancement of the Cascade-RCNN with attention mechanism and deformable convolution to detect hub defects; Liu et al*.*^22^ proposed an improved anchor-based RetinaNet algorithm for detecting power line defects in drone images.

Unlike anchor-based object detectors, anchor-free object detectors perform prediction and regression on pixels directly without requiring the predefinition of anchor boxes. Representative networks in this category include CenterNet^27^ based on key point detection, FCOS^25^ based on center point regression, and YOLOX^28^. Since anchor-free methods do not require prior design of anchors, they have a simplified model structure and reduced hyperparameter settings. Recently, anchor-free detectors have been used for defect detection. For instance, Wang et al*.*^29^ proposed the CenterNet-CL model with an hourglass backbone for defect detection in additive manufacturing. Tian et al*.*^30^ incorporated extended convolutions and the CIOU loss function into the CenterNet to optimize the detection of surface defects on steel strips. Long et al*.*^24^ proposed the FCOS-ITN model, which achieved an accuracy of 90.27% on a plastic packaging defect dataset. Yu et al*.*^26^ designed an improved anchor-free defect detection network based on FCOS (called CABF-FCOS) and proposed a bidirectional feature fusion network by incorporating a channel attention mechanism (CAM) into the feature extraction network. This network achieved a 3.33% higher average precision than FCOS. Han et al*.*^31^ introduced CAM and the SIoU loss function into the YOLOX model for overhead insulator detection.

Though the above-mentioned object detectors have shown promising performance in defect detection, anchor-based detectors heavily rely on anchors, resulting in limited detection generality. Meanwhile, the anchor setting introduces excessive hyperparameters and thus brings about high computational overhead. A matching imbalance between positive and negative samples caused by excessive anchors may lead to suboptimal detection results. By contrast, anchor-free detectors have high detection efficiency and low computational overhead but still have several defects. Pixel-wise regression methods are highly dependent on the accuracy of extracted feature information and may suffer from misalignments during feature fusion, resulting in false positives and false negatives, which in turn affect the detection ability of defect detection methods. To this end, this study proposes an improved anchor-free network (denoted as DLA-FCOS) with optimized feature extraction and fusion structures to enhance the accuracy of defect detection in various industrial products.

### Dual-branch feature extraction

Most defect detection tasks rely on multi-scale features extracted by the backbone network, so the choice of the feature extraction network is crucial for the accuracy and effectiveness of detectors. Currently, various well-established feature extraction networks (e.g., MobileNet^32^, ResNet^33^, GhostNet^34^, and TinyNet^35^) have been applied to visual tasks. Meanwhile, great efforts have been dedicated to improving the internal structure of feature extraction networks to better suit defect detection tasks. For instance, Zhang et al*.*^36^ proposed to replace the YOLOv5s in defect detection of wind turbines with MobileNetv3 to improve the computation speed of the model; Zhou et al*.*^37^ introduced a novel backbone architecture called LGB-Net for industrial defect detection.

Most defect detection networks have single-branch backbone structures, and after feature extraction, multi-scale features of defect images are produced. However, surface defects of industrial products are characterized by complex textures and non-dense small target defects, and existing feature networks cannot extract the feature information of these defects. Therefore, they are not suitable for surface with scattered distribution and complex texture of defects. In addition, large models can address accuracy degradation issues at the expense of increased training burdens. Therefore, a lightweight network that can accurately extract complex features is urgently needed. In recent years, dual-branch fusion networks have been applied to image analysis. For example, Yao et al*.*^38^ proposed a dual ViT based on the Transformer framework, which extracts image feature information and semantic information separately and concatenates the extracted information to enhance local feature information. To accurately extract complex defects in industrial production, this study introduces a dual-branch feature extraction network that acquires multi-scale features through dual-branch information fusion and feature information supplement to accurately detect multiple types of complex defects.

### Feature misalignment

Feature misalignments usually occur in the mapping of information of different scales. Most anchor-free networks fuse multi-scale features by using the Feature Pyramid Networks (FPN) to guarantee scale invariance in object detection. However, the direct fusion of local features may lead to severe feature misalignment as learnable parameters are absent in upsampling methods^39^, resulting in incorrect classification of defect types and ultimately affecting the detector accuracy. For convolutional networks, additional modules are introduced to address the issue of feature misalignment in most cases. For instance, deformable convolution networks (DCN) are incorporated to maintain feature invariance^40^. Based on this, FaPN^41^ proposed feature alignment using DCN during upsampling and introduced attention mechanisms for channel selection in the information of the original scale. In guided anchoring^42^ and AlignDet^43^, DCN was added to anchor-based networks to handle feature misalignment, and improved accuracy was achieved. Most of these networks aim to optimize the performance of anchors, with limited effects on anchor-free detectors. The closest anchor-free work comes from AlignPS^44^, where feature misalignment was eliminated by improving the head structure of FCOS and incorporating deformable convolution. Nevertheless, this detector was designed for pedestrian prediction and crowd search tasks and is not suitable for detecting non-dense defects such as defects in the cut layer of tobacco packets. The above improved methods significantly improve the detectability of specific tasks. However, DCN requires a large number of parameters, imposing a huge training burden. For example, in FaPN^41^, the FPN designed based on DCN increases the number of parameters by 4.5 M compared to the baseline, accounting for 15% of the network. To eliminate feature misalignment in the detection of defects and reduce the complexity of the model, this study proposes the strategy LA-RFPN based on local feature enhancement and self-attention guidance.

## Method

### Overall architecture

**Figure 1:**
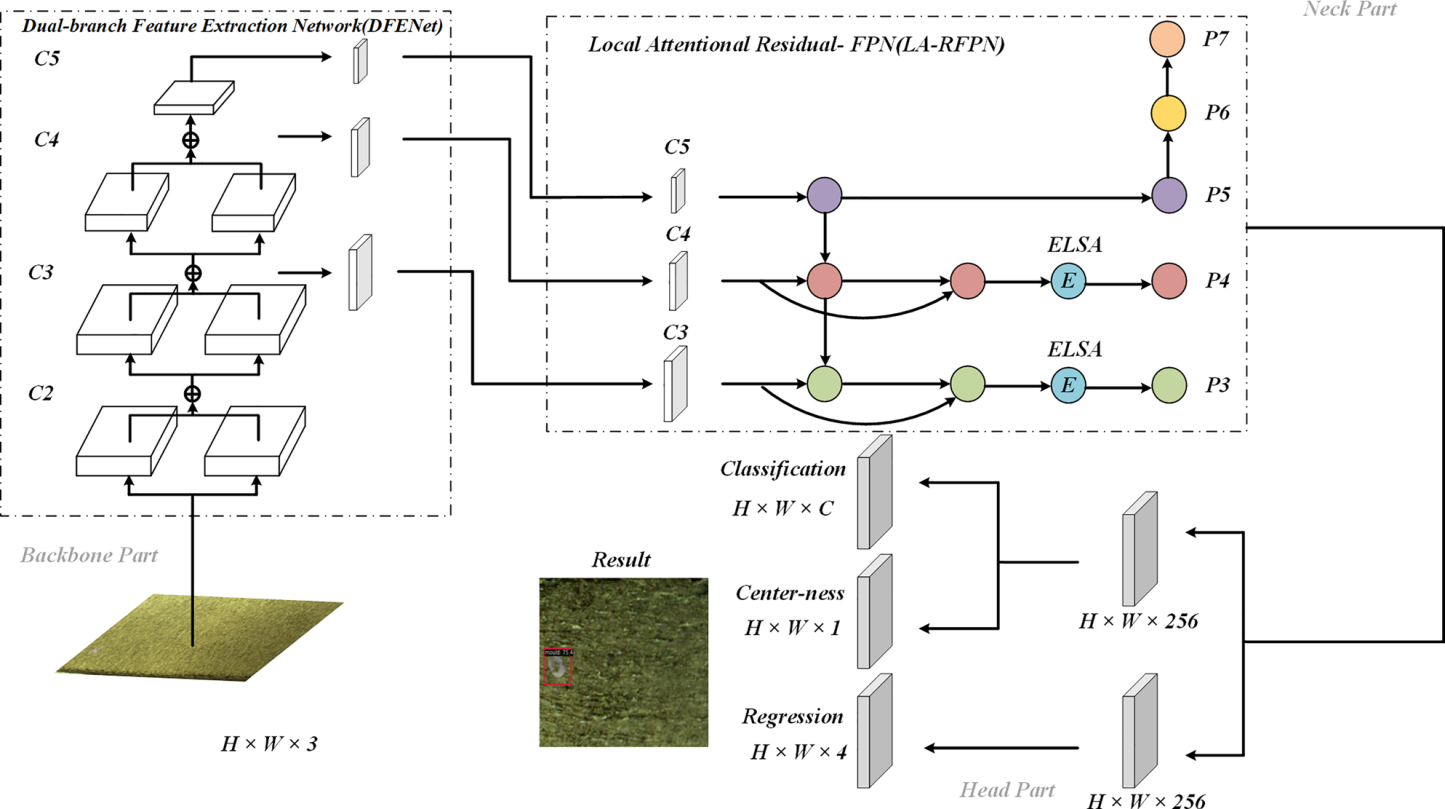
The structure of the DLA-FCOS network. Contains the detailed structure of DFENet and LA-RFPN.

To enhance the precision of anchor-free defect detection and reduce false positives and false negatives, this study proposes an improved dual-branch local attention network called DLA-FCOS (Dual-branch Local Attention FCOS). The architecture of DLA-FCOS is shown in Fig. 1. It can be seen that DLA-FCOS retains the FCOS network prediction layer framework but reconstructs the backbone and neck parts. Specifically, in the backbone part, a lightweight Dual-branch Feature Extraction Network (DFENet) is proposed, which can enhance accuracy while maintaining detection performance. In the neck part, a novel architecture called Local Attentional Residual-FPN (LA-RFPN) is developed to address the feature misalignment issue of FPN. First, local features are enhanced through residual connections and multi-scale fusion to mitigate the effects of offsets generated by upsampling and downsampling. Then, the Embedded BiLevel Routing Attention (ELSA) is proposed and applied to the LA-RFPN network to filter out invalid features from offset positions, further mitigating the offset issue.

As illustrated in Fig. 1, the RGB images containing non-dense defects (such as the cut layer of tobacco packets) are fed into the backbone module to extract multi-scale features of the images. Features are extracted from layers C3, C4, and C5 of the feature extraction network and then input into LA-RFPN for feature enhancement fusion; the fused features are further processed by ELSA to remove offsets, thus obtaining more accurate feature images (P3 and P4). C5 is processed by a 3 × 3 convolution to obtain P5, and then P5 is downsampled twice to generate P6 and P7. Finally, the prediction layer uses the extracted P3-P7 feature images to identify and locate defects.

### Dual-branch feature extraction network (DFENet)

**Figure 2:**
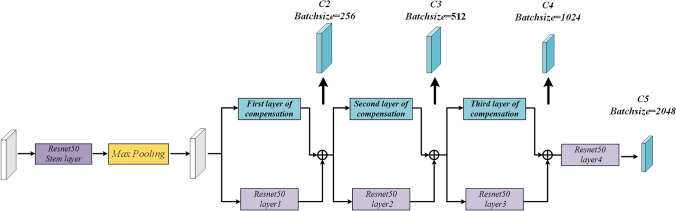
The overall architecture of the backbone network in DLA-FCOS.

The FCOS network^25^ uses Resnet-50 for feature extraction. However, since the images have complex defect textures and abundant background noise, conventional backbone networks will have poor feature extraction performance on the images, which further affects the subsequent fusion and prediction tasks. Deep networks (e.g., VGG^45^) can overcome the limitations of conventional networks in feature extraction, but their complex structures lead to slower computation speeds. Additionally, different defects in the surface pose varying detection difficulties. As a result, the use of the backbone to pursue higher accuracy is prone to excessive feature extraction, resulting in false positives. To address these issues, this study proposes a dual-branch feature extraction network. The overall architecture of this network is demonstrated in Fig. 2.

**Figure 3:**
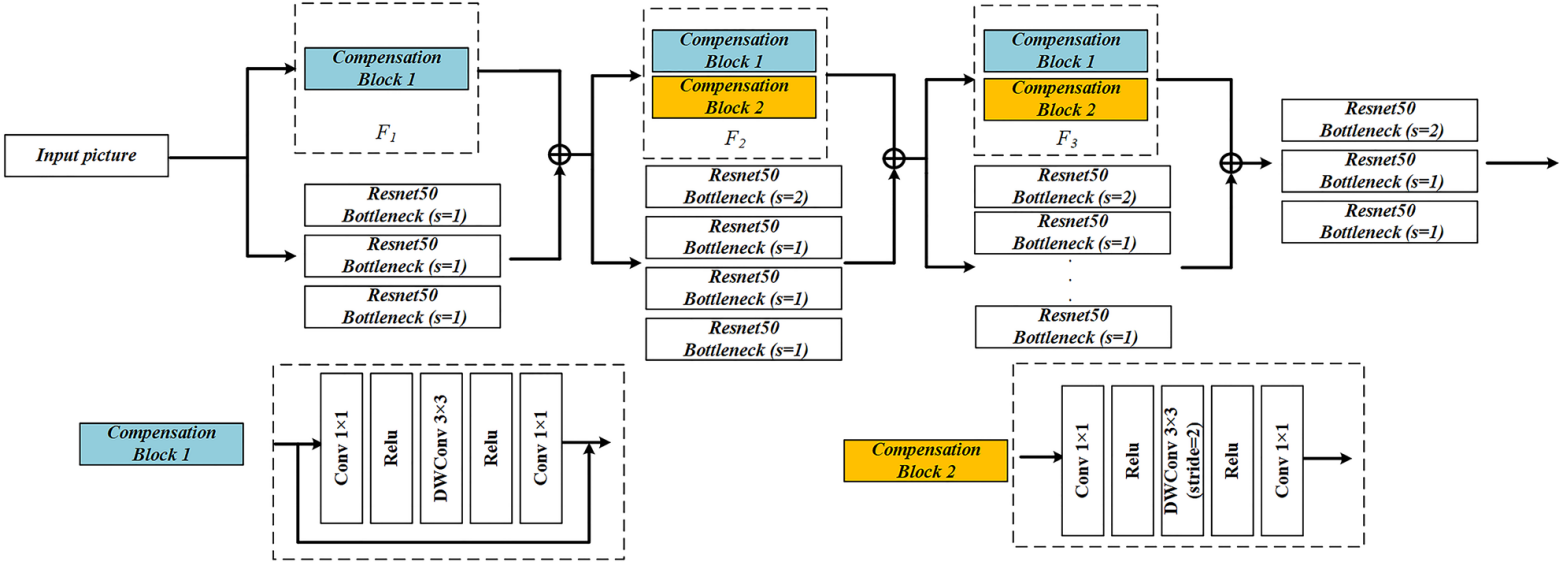
Dual-branch Feature Extraction Network.

Compared to the Resnet-50 network, DFENet adopts a dual-branch parallel fusion extraction method involving a main branch and a compensation branch. In this method, the extracted multi-scale features are added in a pixel-wise manner to facilitate feature information interaction. As shown in Fig. 3, the main branch has the same structure as Resnet and is utilized to extract the feature information of images, while the compensation branch consists of three layers F_1_, F_2_, and F_3_, with each layer composed of multiple Compensation Blocks, and is used to extract the feature information of complex defects. Herein, F1 consists of one Compensation Block 1, while F2 and F3 consist of one Compensation Block 1 and one Compensation Block 2. This study introduces depth-wise separable convolution (DWConv)^46^ into the Compensation Blocks to replace normal convolutions. DWConv can obtain higher computational efficiency at the expense of reduced accuracy, thereby leading to a lightweight design for the compensation branch. The specific process is described as follows: First, the pooled input images are extracted by resnet layer1 and F1 respectively, and the low-level feature image C2 is obtained after fusion. Then, the superimposed feature images are extracted in parallel with compensation branches and multiple Resnet blocks to obtain C3 and C4. Finally, the high-level feature image C5 is obtained by further enriching the semantic information through resnet layer4. The features obtained after dual-branch fusion contain multi-scale pixel information, which allows for an expansion of the receptive field while minimizing the loss of positional information, thereby enabling the precise extraction of complex defects.

### Local attentional residual-FPN (LA-RFPN)

**Figure 4:**
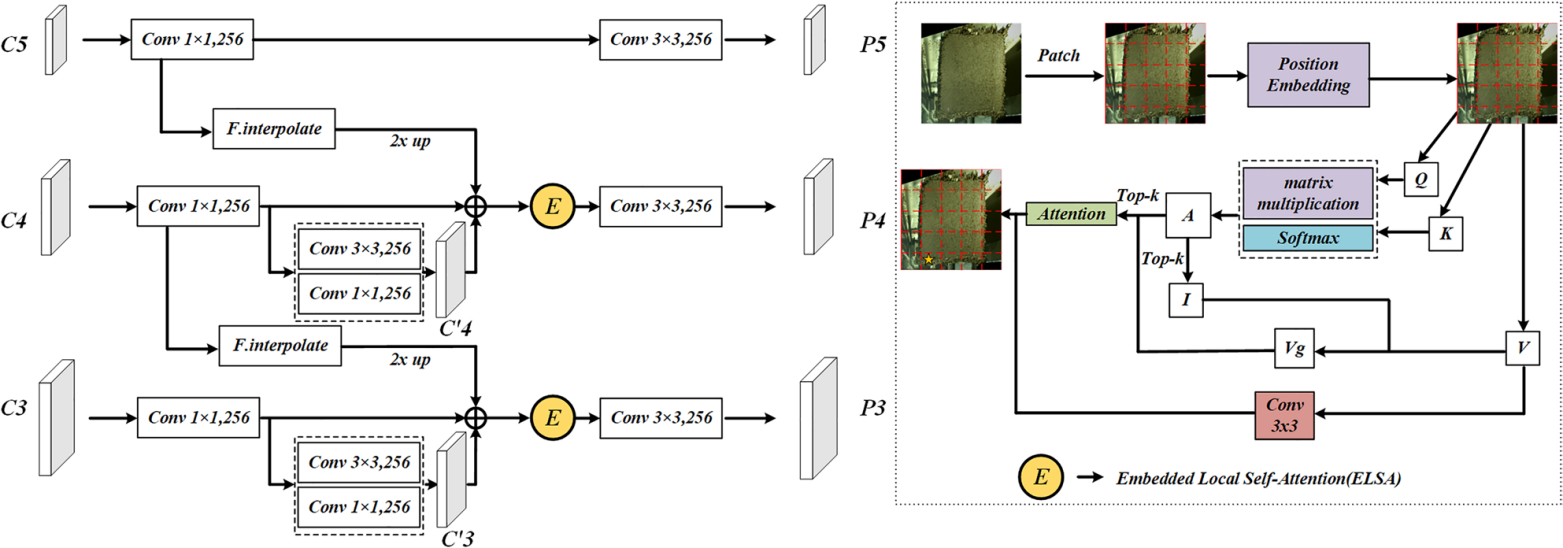
The framework of Local Attentional Residual-FPN.

Since foreign objects or defects are relatively small, they are susceptible to background noise, so more precise multi-scale feature fusion is required. FPN directly upsamples high-level features and adds them to low-level features. However, the significant scale change may result in serious feature misalignment during the feature fusion process, bringing about false positives. To address this issue, this study proposes a novel FPN structure called Local attentional Residual-FPN (LA-RFPN, see Fig. 4) for detecting non-dense defects such as tobacco packets.

To address the feature misalignment issue during multi-scale feature fusion, this study reconstructs and optimizes the FPN structure. As illustrated in Fig. 4, the features extracted from the last three stages of the backbone are fused, and the feature images are denoted as {C3, C4, C5}, with scales of 1/8, 1/16, and 1/32, respectively. After the feature images are fed into the fusion network, they first undergo a 1 × 1 convolution to fix the channel dimension to 256. Then, C3 and C4 undergo a 3 × 3 convolution and a 1 × 1 convolution respectively to obtain semantically enriched feature images C’3 and C’4. Subsequently, C’3 and C’4 are connected with C3 and C4 through residual connections and then added with the upsampled features C4 and C5 respectively to form a three-branch structure. Compared with FPN which directly adds upsampled features, the three-branch structure not only enhances the semantic information of local features but also preserves essential information during multiscale fusion while avoiding the loss of lower-level feature information. Since the high-level feature C5 already contains rich feature information, it is not further enhanced. Finally, the added enhanced features are fed into the ELSA to further eliminate misalignment during multi-scale fusion. The details are provided in the following sections.

The self-attention mechanism is widely used in Transformer, and it focuses on key feature information while paying less attention to irrelevant information. However, self-attention is generally used to enhance convolutional expression in convolutional networks^47^. To further eliminate the misalignment feature information and mitigate the impact of feature misalignment on detection, inspired by BiFormer^48^, this study proposes an attention mechanism called ELSA suitable for CNNs to eliminate false information during multi-scale fusion. ELSA can take local features and multi-scale features with enhanced features into account and ignore misalignment caused by upsampling. To adapt to the convolutional network architecture, this study introduces learnable position encoding into feature images ${\text{X}}\in {\mathbb{R}}^{H\times W\times C}$ in the ELSA:

1 $$\begin{array}{c}{{\text{X}}}_{E}=X+{{\text{X}}}_{P}\end{array}$$

where ${{\text{X}}}_{P}\in {\mathbb{R}}^{H\times W\times C}$ and it has the same size as feature images, with *H*, *W*, and *C* representing the length, width, and channels of the input feature images, respectively; ${{\text{X}}}_{E}\in {\mathbb{R}}^{H\times W\times C}$ is the feature image to be embedded.

According to the sizes of the input feature images, ${{\text{X}}}_{E}$ is partitioned into non-overlapping regions of size N × N. For the defect dataset in the cut layer of tobacco packets, N = 5. The partitioned ${{\text{X}}}_{E}^{d}\in {\mathbb{R}}^{{N}^{2}\times \frac{W\times H}{{N}^{2}}\times C}$. The linear mappings for a query Q, key K, and value V are derived as follows:

2 $$\begin{array}{c}Q={{\text{X}}}_{E}^{d}{{\text{W}}}^{q},K={{\text{X}}}_{E}^{d}{{\text{W}}}^{k},V={{\text{X}}}_{E}^{d}{{\text{W}}}^{v},\end{array}$$

where ${{\text{W}}}^{q}$, ${{\text{W}}}^{k}$, and ${{\text{W}}}^{v}$ represent the weight of a query, key, and value, respectively.

According to the vector value for each partition region (i.e., $\frac{W\times H}{{N}^{2}}$), Q and K are averaged to obtain the region-level query ${{\text{Q}}}^{r}$ and key ${{\text{K}}}^{r}\in {\mathbb{R}}^{{N}^{2}\times C}$. Then, transpose matrix multiplication is performed to obtain the adjacency matrix ${{\text{A}}}^{r}\in {\mathbb{R}}^{{N}^{2}\times {N}^{2}}$ that indicates the similarity between regions. Meanwhile, the top-k value is set to obtain k regions most relevant to the region and their position indices:

3 $$\begin{array}{c}{{\text{A}}}^{r}={{\text{Q}}}^{r}{{({\text{K}}}^{r})}^{T},{{\text{I}}}^{r}=topindex{({\text{A}}}^{r}),\end{array}$$

Since the RFPN enhances local features, offsets are more likely to be considered irrelevant regions and ignored, which can help eliminate feature misalignment. Additionally, the calculated ${{\text{I}}}^{r}$ is used to guide the combination of relevant keys and values to output the results of multi-head attention as *P*:

4 $$\begin{array}{c}{{\text{K}}}^{g}=gather\left({\text{K}},{{\text{I}}}^{r}\right),{{\text{V}}}^{g}=gather\left({\text{V}},{{\text{I}}}^{r}\right),{{\text{K}}}^{g},{{\text{V}}}^{g}\in {\mathbb{R}}^{{N}^{2}\times \frac{kWH}{{N}^{2}}\times C}\end{array}$$

5 $$\begin{array}{c}P=Attention\left(Q,{{\text{K}}}^{g},{{\text{V}}}^{g}\right)+{Conv}_{3x3}(V)\end{array}$$

The value is added to the attention through a 3 × 3 convolution for local enhancement^49^. Subsequent experiments demonstrate that applying ELSA to FPN can improve the accuracy of detecting defects such as tobacco packet defects.

## Experiments and results

### Experiment dataset

**Figure 5:**
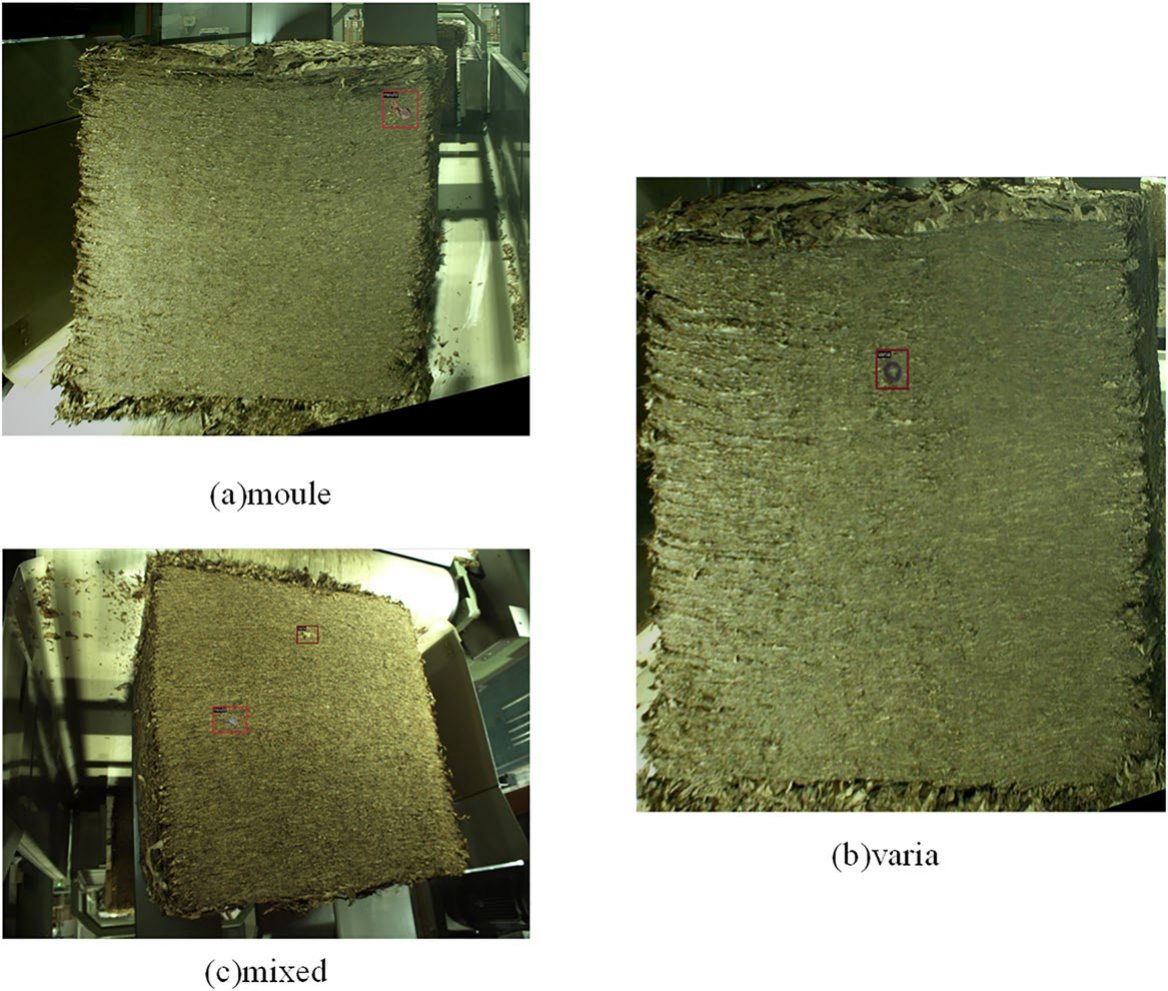
Visualization of defects of different types in CLTP-DD.

To validate the methods proposed in this study, the cut layer of tobacco packet defect dataset (CLTP-DD)^50^ is used. Since the number of foreign object samples collected is relatively limited, this study artificially increases the number of foreign object samples and performs image fusion to enable the object detection algorithm to learn sufficient features. As shown in Fig. 5, the foreign objects in the dataset images mainly include paper, hemp rope, metal, and moldy tobacco, and there are two types of defects: moule and varia. After the images are fused, 4118 pieces of defect data are obtained, and there are 4494 foreign objects in total, which are categorized into two types: mold and variation. The image size is 1000 × 1120 pixels. In the experiments, the dataset is divided into a training set and a testing set at a ratio of 7:3, where the training set is used for training and fine-tuning networks and the testing set is used for testing network performance.

### Experimental platform

The experimental platform is a server equipped with an Intel(R) Xeon(R) Gold 6330 CPU @ 2.00 GHz and an RTX 3090 (24 GB video memory) GPU, and the server runs Ubuntu 20.04 operating system and Cuda 11.3. The proposed FRN uses pre-trained weights from ResNet50, while other modules are initialized with a standard deviation of 0.001. Before training, all input images are resized, and multi-scale training is conducted to resize the size of the original input images from 1000 × 1120 to 640 × 640 and 800 × 800. During training, a pre-training model trained on ImageNet is used to initialize the ResNet50 backbone, and SGD (Stochastic Gradient Descent) with a momentum of 0.9 and weight decay of 0.001 is employed to optimize the entire network. In the training process, a fine-tuning strategy is applied. Specifically, the model is first trained for 24 epochs with a learning rate of 10^–3^ and a batch size of 2, and then it is further trained for 36 epochs, with the weight from the last epoch of the first step as the pre-training weight, an initial learning rate of 10^–3^, and a batch size of 2, and the learning rate gradually decays to 10^–4^. All experiments are implemented using PyTorch 1.11.0 on the MMDetection platform.

### Evaluation metric

In this study, precision, recall, average precision (AP), Frames per second (FPS), Params, and giga floating-point operations per second (GFLOPs) are used as evaluation metrics for DLA-FCOS:

$$\begin{array}{c}P=\frac{TP}{TP+FP}\end{array}$$

$$\begin{array}{c}R=\frac{TP}{TP+FN}\end{array}$$

$$\begin{array}{c}AP=\frac{{\int }_{0}^{1}P{\text{d}}R}{N}\end{array}$$

where TP and FN represent the correct and incorrect identification of all defects, respectively; FP represents the number of non-defects that are incorrectly identified. AP represents the average detection accuracy of the model on the test set. AP_50_ represents the AP at IoU = 0.5. In this study, the mean value mAP of AP_50_ of different types of defects is calculated and adopted to evaluate the overall detection performance of the proposed model. FPS represents the number of images the target network can detect per second, which is used to measure the inference efficiency of the model. Params and GFLOPs measure the complexity of the model.

### Detection result analysis on the CLTP-DD

To verify the performance of DLA-FCOS on the CLTP-DD, ablation experiments are conducted, and the experimental results are compared with those of mainstream object detectors. The details are as follows.

#### Ablation experiments

According to the above-mentioned three innovative points, ablation experiments are conducted, and the results are listed in Table 1. It can be seen that, after DFENet is used as the backbone, mAP improves by 2.7% compared to the baseline network, and the detection performance of the varia defect reaches 95.3% in the case of a large proportion of complex defects, demonstrating the precise feature extraction capability of DFENet for complex features. To eliminate the impact of the feature extraction network, experiments are also conducted by improving the FPN only. After RFPN and ELSA are introduced, the mAP increases by 1.9% and 2.5% respectively. After applying the LA-RFPN structure, the overall Params only increase by 1.6 M. The results indicated that the proposed method that enhances local features and combines self-attention effectively addresses the feature misalignment issue. Additionally, experiments are carried out on DLA-FCOS, where the mAP reaches 96.8%, which is 3.0% higher than that of the FCOS.

**Table 1:** Ablation studies on the detection part of the CLTP-DD.

Network	Backbone	RFPN	ELSA	Mould (%)	Varia (%)	mAP (%)	Params (%)	GFLOPs
FCOS	Resnet50			96.0	91.7	93.8	32.13	126.0
DLA-FCOS(Ours)	DFENet			97.8	95.3	96.5	39.43	166.0
	Resnet50	√		96.3	95.0	95.7	33.45	134.1
	Resnet50	√	√	96.6	96.0	96.3	33.71	146.4
	DFENet	√	√	97.2	96.5	96.8	41.01	186.4

#### Comparison with mainstream object detection algorithms

To demonstrate the advantages and effectiveness of the proposed method, it is compared with the mainstream networks Faster R-CNN^51^, SSD, YOLO V3^52^, YOLO V5, RetinaNet, YOLOX, DINO^53^, Mask R-CNN, SwinTD^54^, and FCOS on the CLTP-DD. The results are shown in Table 2. In terms of mAP, DLA-FCOS outperforms other types of networks, with a 0.4% improvement over the state-of-the-art (SOTA) network SwinTD. In terms of testing time and speed, DLA-FCOS slightly underperforms the baseline network but is comparable to RetinaNet, and it can meet real-time defect detection requirements for tobacco packets. Overall, the proposed DLA-FCOS network can effectively detect foreign objects in tobacco packets during production.

**Table 2:** Experimental results on the CLTP-DD.

Network	Backbone	Params (M)	GFLOPs	mAP (%)	FPS (img/s)
Faster R-CNN	ResNet50	164.96	379.69	78.7	5.4
SSD	VGG16	24.53	87.86	93.8	28.3
YOLO V3	Darknet53	61.53	193.87	93.6	12.8
YOLO V5-L	CSPDarknet53	46.14	53.98	94.2	37.6
RetinaNet	Swin-T	36.84	210.29	80.5	17.2
Deformable DETR	ResNet50	39.82	195.23	95.1	10.1
YOLOX-S	CSPDarknet53	8.94	33.3	91.3	16.9
DINO	ResNet50	47.54	197.00	95.3	25.1
Mask R-CNN	Swin-T	47.38	261.81	95.6	9.7
SwinTD	Swin-T	47.38	262.9	96.4	9.6
FCOS	ResNet50	32.13	125.95	93.8	22.0
DLA-FCOS(Ours)	DFENet	41.01	186.37	96.8	20.7

### Detection result analysis on public datasets

#### Experiment public dataset

**Figure 6:**
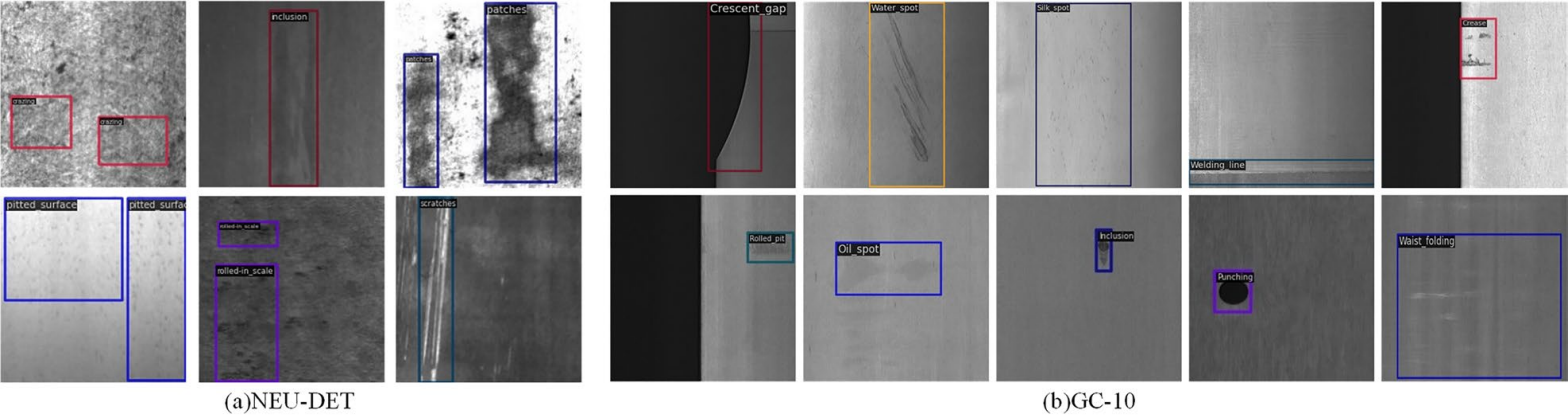
Visualization of defects of different types in NEU-DET and GC10-DET.

To further validate the effectiveness of DLA-FCOS, experiments are carried out on the NEU-DET^55^ and GC10-DET datasets. NEU-DET includes 6 different types of steel strip defects, namely rolled-in scale, patches, crazing, pitted surface, inclusion, and scratches. Each defect contains 300 images, and there are 1800 images in total. Unlike CLTP-DD, the 1800 images are divided into a training set, a validation set, and a testing set at a ratio of 7:2:1. GC10-DET mainly includes 10 common steel surface defects, namely punching (Pu), welding line (Wl), crescent gap (Cg), water spot (Ws), oil spot (Os), silk spot (Ss), inclusion (In), rolled pit (Rp), crease (Cr), and waist folding (Wf). GC10-DET contains 2294 defect images, which are divided into a training set and a testing set at a ratio of 8:2. The defects of the above datasets are shown in Fig. 6.

#### Experiment results and comparison analysis

To comprehensively evaluate detection performance, accuracy metrics at different IoU thresholds, including AP_bbox_, AP_50_, and AP_75_ are used for evaluating DLA-FCOS. Ablation experiments are conducted on NEU-DET in the same way as CLTP-DD, and the experimental results are listed in Table 3.

**Table 3:** Experimental results on public datasets.

Datasets	Network	Backbone	RFPN	ELSA	AP_50_	AP_75_	AP_bbox_
NEU-DET	FCOS	Resnet50			75.2	30.5	36.8
	DLA-FCOS(Ours)	DFENet			77.4	28.5	36.8
		Resnet50	√		76.1	31.9	37.2
		Resnet50	√	√	77.0	31.7	36.6
		DFENet	√	√	78.4	34.7	38.1
GC10-DET	FCOS	Resnet50			60.5	19.4	27.2
	DLA-FCOS(Ours)	DFENet	√	√	67.7	30.3	33.2

As shown in Table 3, the ablation experiment results of DLA-FCOS on NEU-DET are consistent with those on CLTP-DD. After the integration of relevant modules, the precision is significantly higher than that of the baseline network. Specifically, AP_50_, AP_75_, and AP_bbox_ increase by 3.2%, 4.2%, and 1.3% respectively compared with those of the baseline network, with AP_50_ reaching 78.4%, demonstrating excellent performance on this dataset. In GC10-DET, AP_50_ increases significantly by 7.2% compared with that of FCOS, demonstrating the effectiveness of the proposed improved module.

To reflect the generalization of DLA-FCOS, it is compared with the SOTA defect detector on NEU-DET and GC10- DET. As illustrated in Table 4, the proposed DLA-FCOS achieves slightly lower accuracy compared to the anchor-based SOTA detector but outperforms anchor-free detectors (e.g., CABF-FCOS, SAPD). Especially, DLA-FCOS excels in detecting inclusion defects compared to other defect detectors. As shown in Table 5, DLA-FCOS achieves the highest accuracy in detecting defects such as punching, with an mAP of 67.7%, which is slightly lower than that of MSC-Dnet but is higher than that of other SOTA networks. It is noteworthy that most of the aforementioned SOTA networks are designed for the NEU-DET and GC10-DET detection tasks, while DLA-FCOS is designed for CLTP-DD and achieves good results on these datasets. Therefore, DLA-FCOS has great effectiveness and generalization capability in defect detection tasks.

**Table 4:** Results on the NEU-DET dataset compared with SOTA defect detectors.

Method	Crazing	Inclusion	Patches	Pitted surface	Rolled in scale	Scratches	mAP (%)
EDDN^56,56^	41.7	76.3	86.3	85.1	58.1	85.6	72.4
CABF-FCOS^26,26^	55.4	75.0	93.5	88.9	62.9	84.4	76.7
kou's YOLOv3^57,57^	38.9	73.7	93.5	74.8	60.7	91.4	72.2
MSFT-YOLO^58,58^	56.9	80.8	93.5	82.1	52.7	83.5	75.2
DEA_RetinaNet^59,59^	60.9	82.5	94.3	95.8	67.2	74.1	79.1
SAPD^60^	60.9	82.5	93.3	87.4	42.9	97.8	73.2
MSC-Dnet^11^	42.4	84.5	94.3	91.5	71.6	92.0	79.4
DLA-FCOS (Ours)	56.7	89.5	91.2	83.7	65.9	83.2	78.4

**Table 5:** Results on the GC10-DET dataset compared with SOTA defect detectors.

Method	Pu	Wl	Cg	Ws	Os	Ss	In	Rp	Cr	Wf	mAP (%)
DCC-CenterNet^30^	84.4	85.5	96.2	77.3	50.9	54.8	30.2	13.9	49.9	76.6	61.9
EDDN^56,56^	90.0	88.5	84.8	55.8	62.2	65.0	25.6	36.4	52.1	91.9	65.1
Wang’s YOLOv3^61^	62.8	59.7	85.1	68.6	27.2	30.7	8.9	1.5	16.4	50.3	41.1
MSC-DNet^11^	95.5	96.1	94.9	76.5	66.5	65.8	34.1	53.4	48.5	84.0	71.6
DLA-FCOS (Ours)	96.5	92.4	94.6	81.0	66.8	65.2	40.1	27.8	31.9	77.6	67.7

### Visualizations

**Figure 7:**
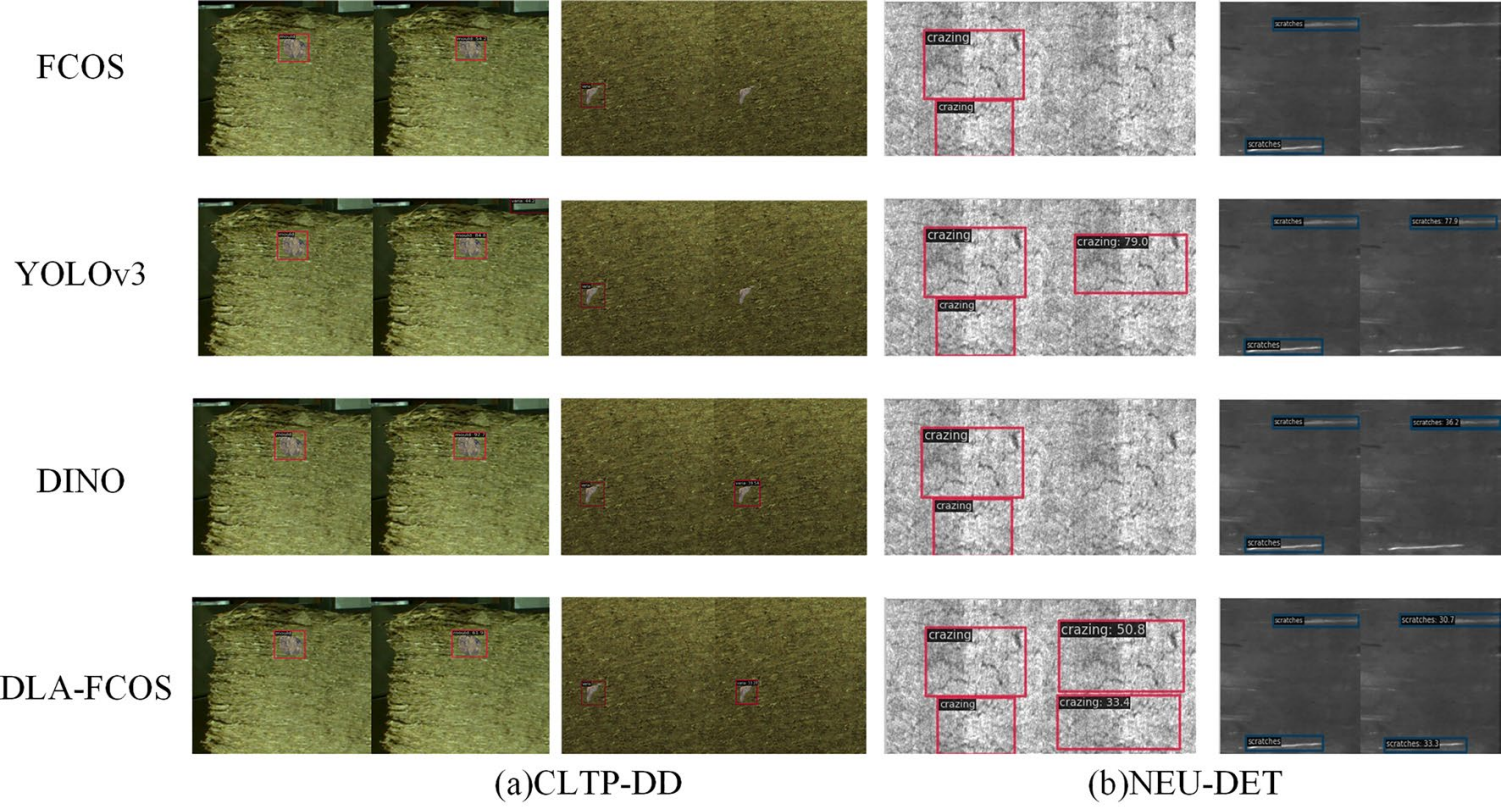
Visualization of detection results. Each picture is composed of two original images. The left side of the picture is the superposition of the real box and the original image, and the right side is the superposition of the prediction box and the original image.

To verify that DLA-FCOS can effectively mitigate false positives and false negatives in the defect detection process, visualizations are carried out on CLTP-DD and NEU-DET testing sets for the anchor-free detector FCOS, anchor-based detector YOLOv3, Transformer-based DINO, and the proposed DLA-FCOS (see Fig. 7). It can be seen that, in the CLTP-DD detection tasks, FCOS and YOLOv3 detectors yield false positives and false negatives; in contrast, DLA-FCOS and DINO are more suitable for detecting a single type of defect. In the NEU-DET detection tasks, the DLA-FCOS has a less significant confidence level compared with other networks, but it performs better in non-dense and multi-defect detection tasks, and it locates defects more accurately than the FCOS.

## Discussion

### Effectiveness analysis of DFENet

A lightweight design is used in the branches of DFENet to reduce the load of parameter training. The ablation experiments in Section "Experiments and results" make evaluation only in accuracy, which cannot fully demonstrate that DLA-FCOS can detect complex defects rapidly and accurately. In view of these, the GFLOPs, Params, FPS, and accuracy of the backbone are considered (see Table 6).

**Table 6:** Comparison of time complexity and space complexity between ResNet50 and DFENet on CLTP-DD.

Backbone	Params (M)	GFLOPs	mAP (%)	AP_s_ (%)	AP_m_ (%)	AP_l_ (%)	FPS
ResNet50	32.13	125.95	93.80	21.8	24.4	40.6	22
DFENet	39.43	166.00	96.50	32.0	44.4	42.0	21

**Figure 8:**
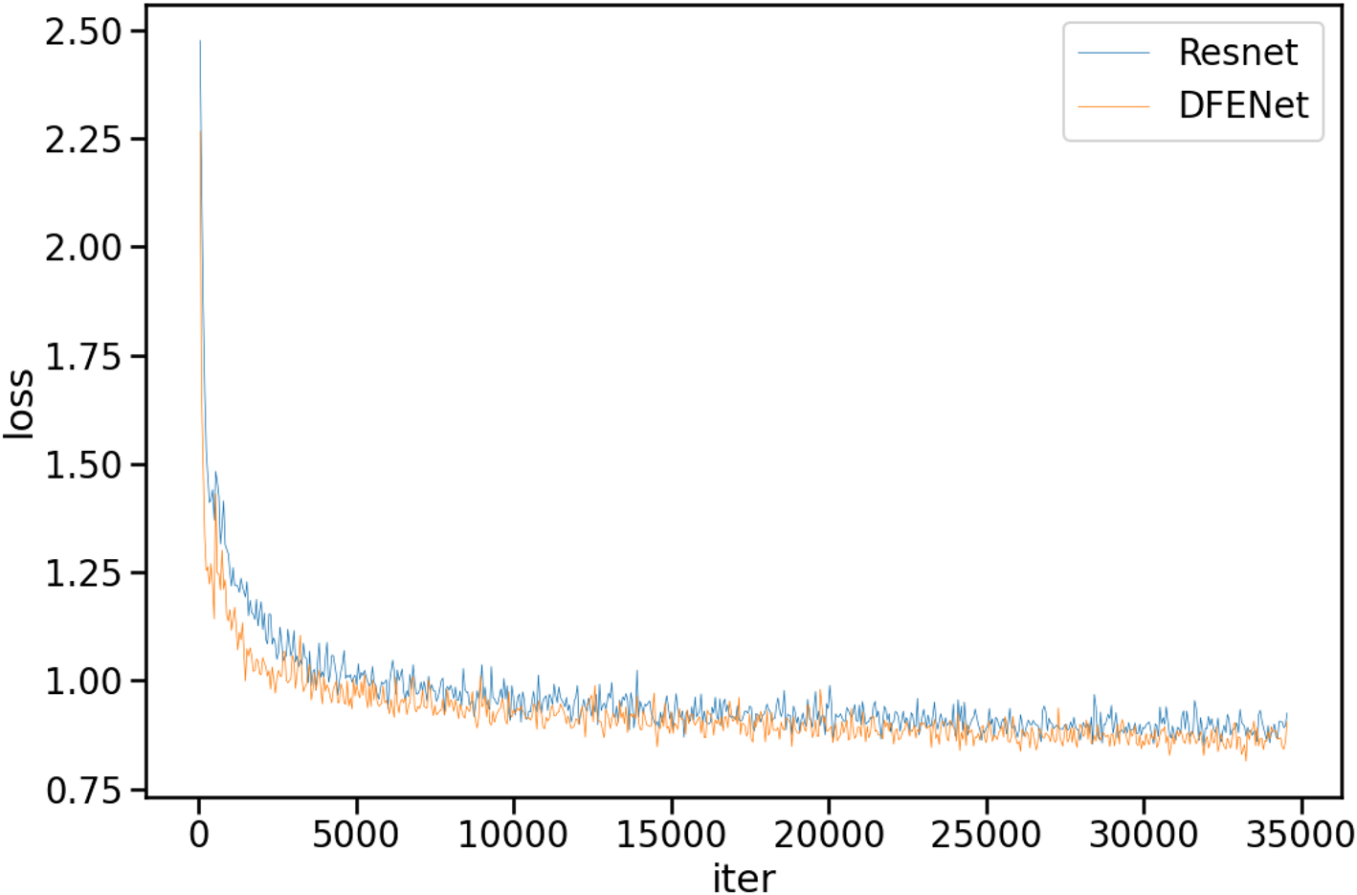
Loss comparison of different backbones on the CLTP-DD.

Herein, AP_s_ denotes the small target defect accuracy under area < 32^2^ px, AP_m_ denotes the middle target defect accuracy under 32^2^ px < area < 96^2^ px, and AP_l_ denotes the large target defect accuracy under 96^2^ px < area. It can be seen from Table 6 that DFENet exhibits significantly better detection performance for small target defects than the baseline network, with a performance improvement of 11.8%. Meanwhile, it only leads to a 20% increase in parameter load, and the running speed remains almost unchanged, indicating that it can meet the real-time detection requirements in industrial production. Figure 8 shows the loss variations of ResNet50 and DFENet when used as backbones on the CLTP-DD, indicating that DFENet converges faster and performs better.

### Process Analysis of Implementing ELSA

In this study, the ELSA, which is an improved self-attention mechanism, is proposed and used to eliminate feature misalignments. Initially, this study attempted to directly introduce Bi-Level Routing Attention (BRA)^48^ into RFPN, but the experimental results demonstrated a noticeable decline in mAP. It is speculated that the convolutional network lacks guidance by positional encoding, and thus BRA only enriches semantic information during the fusion stage. Hence, based on BRA, this study proposes LA-RFPN by adding learnable position encoding and attention local enhancement, which achieves a detection accuracy of 96.4%.

**Figure 9:**
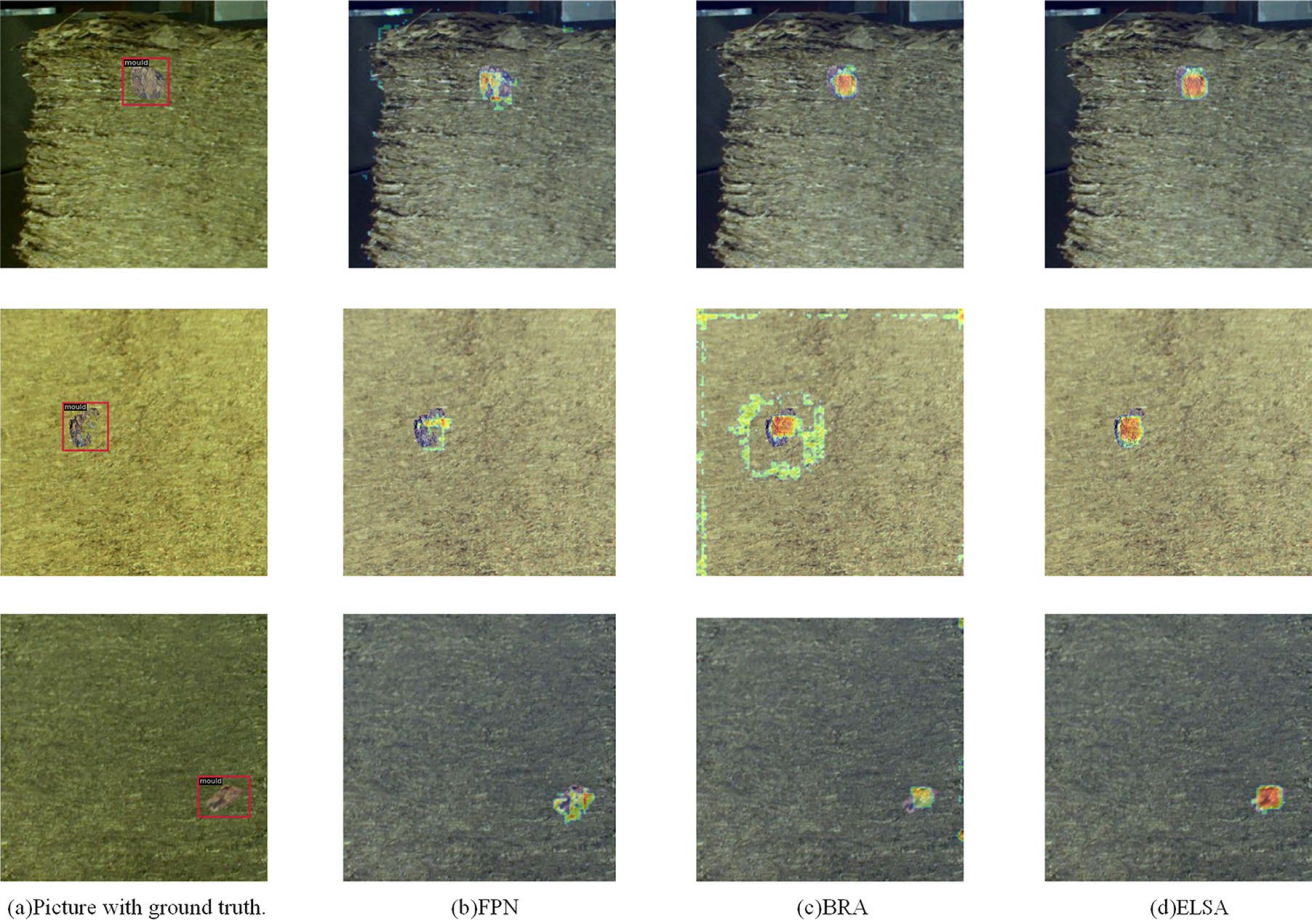
Heatmap visualization. The images are obtained by fusing the original images and heatmaps.

To discuss the performance improvement of ELSA, this study selects samples from the testing set to perform visual feature analysis and sets a heatmap threshold to better visualize areas of interest, as shown in Fig. 9. It can be seen that, due to the lack of supervision by attention mechanisms, the foreground feature distribution in FPN is weak, and many defect features are not contiguous. BRA can focus on significant features but lacks encoding supervision, making it susceptible to background noise interference. By contrast, the proposed ELSA can better locate defect zones, thereby effectively reducing false negatives in the detection process for the cut layer of tobacco packets and improving detection accuracy.

### Analysis of failure cases and future work

**Figure 10:**
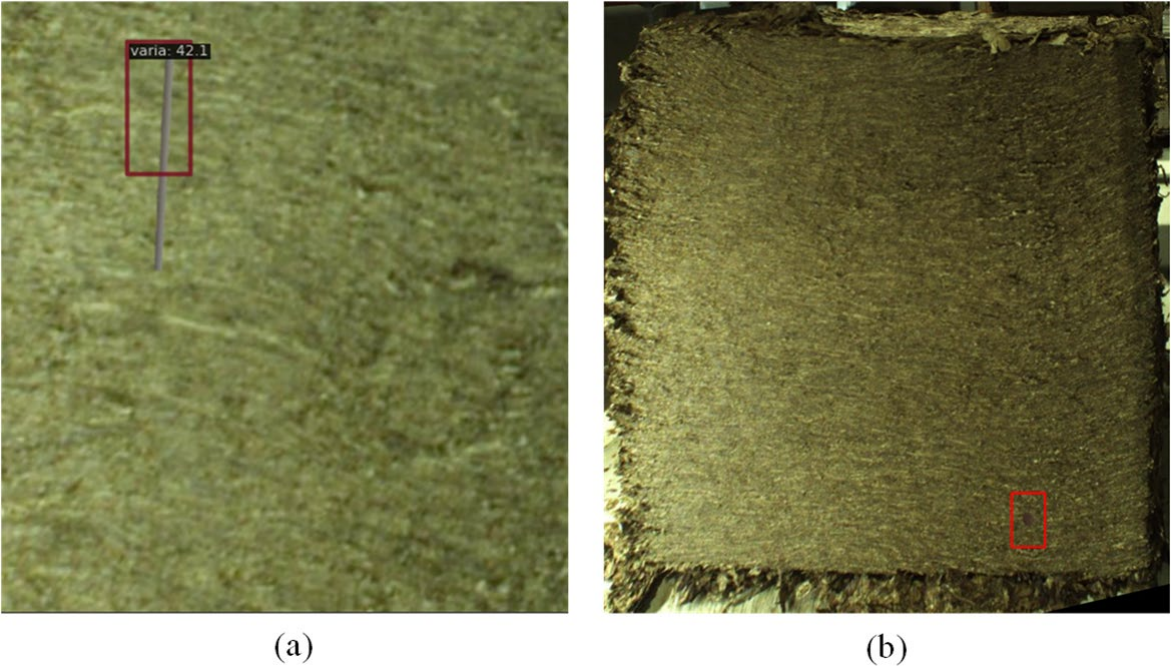
Failure cases.

DLA-FCOS demonstrates excellent performance in the defect detection tasks in the cut layer of tobacco packets but it still has some limitations. In Fig. 10, the red box represents the predicted result, and the number next to the defect indicates the confidence level of the type of defect. As illustrated in Fig. 10a, the defect is not accurately located, and the confidence level is low. This may be due to the large size of the target defect, as a result of which ELSA fails to notice the overall features of the defect. As shown in Fig. 10b, the location of the defect is not detected (the red box in the figure is manually marked). This may be because the foreground information of the defect is highly similar to the background information, leading to a false negative. These cases indicate that the proposed DLA-FCOS still has certain limitations. Future work will optimize the focus mechanism of the attention mechanism and improve the loss function to improve the confidence level of detection. To address the issue of background interference, future studies will consider introducing data enhancement preprocessing algorithms to filter out background noises and reduce false negatives.

## Conclusion

The DLA-FCOS, an anchor-free defect detector, is proposed and applied to the tobacco packet defect detection task with a large proportion of scattered small target defects. First, the DFENet is developed, and complex feature information is extracted by using the dual-branch fusion method. Then, the LA-RFPN is proposed, and the ELSA is introduced to enhance the semantic features of the images and emphasize key features, thereby eliminating the influences of FPN feature misalignment. Additionally, the effectiveness of the DLA-FCOS is validated on the dataset of the cut layer of tobacco packets. The mAP of DLA-FCOS is up to 96.8%, which is 3.0% higher than that of the FCOS. And the mAP of DLA-FCOS is higher than those of Faster R-CNN, Swin-TD, YOLOV5, and DINO. Meanwhile, the generalization capability of the DLA-FCOS is validated on the NEU-DET and GC10-DET datasets. The results indicate that the mAP of DLA-FCOS is respectively 78.2% and 67.7% on the two datasets, which are higher than those of other anchor-free defect detectors. Overall, the proposed DLA-FCOS has good feasibility and high generalization capability, especially for the detection of small defects and complex defects.

## Data Availability

All data generated or analyzed in this study are included in this manuscript. The datasets used in the current study are available from the corresponding author on reasonable request.
